# Zonisamide attenuates morphine tolerance in association with reduced spinal TLR4/p38 MAPK signaling

**DOI:** 10.3389/fphar.2026.1893236

**Published:** 2026-08-26

**Authors:** Li-Ba Gei, Ying-Hua Liu, Hong Guo, Xin Zhao, Lu-Lu Li, Rui Zhang, Hong-Xia Cheng, Juan-Juan Feng, Wei-An Zeng, Yan Yan, Ri-Qi E, Dong-Tai Chen

**Affiliations:** 1 Department of Anesthesiology, Peking University Cancer Hospital (Inner Mongolia Campus)/Affiliated Cancer Hospital of Inner Mongolia Medical University/Inner Mongolia Cancer Center/Inner Mongolia Cancer Hospital, Hohhot, China; 2 Department of Anesthesiology, Key Laboratory of Carcinogenesis and Translational Research (Ministry of Education/Beijing), Peking University Cancer Hospital & Institute, Beijing, China; 3 Department of Anesthesiology, State Key Laboratory of Oncology in South China, Guangdong Provincial Clinical Research Center for Cancer, Sun Yat-Sen University Cancer Center, Guangzhou, China; 4 Department of Anesthesiology, Huizhou Municipal Central Hospital, Huizhou, China

**Keywords:** drug repurposing, morphine tolerance, neuroinflammation, TLR4/p38 MAPK, zonisamide

## Abstract

**Introduction:**

Morphine tolerance limits its long-term clinical utility, and neuroinflammation is a key underlying mechanism. This study investigated whether zonisamide, an antiepileptic drug with anti-inflammatory properties, attenuates morphine-induced neuroinflammation and morphine tolerance.

**Methods:**

In vitro, BV-2 microglial cells were treated with morphine (200 μM) with or without zonisamide (10 μM). Quantitative real-time PCR, enzyme-linked immunosorbent assay (ELISA), and Western blot analysis were used to assess inflammatory mediators and signaling pathways. In vivo, male C57BL/6 mice received repeated morphine injections (10 mg/kg, s.c., twice daily) for 9 days to induce tolerance, with or without zonisamide (30 mg/kg, i.p.). Behavioral tests (hot plate and tail flick) were performed to evaluate analgesic tolerance. Immunofluorescence was performed to assess microglial activation (Iba1), and Western blot analyses were conducted to evaluate TLR4 expression, p38 phosphorylation, and pro-inflammatory cytokine protein levels. Pharmacological inhibition of TLR4 with TAK-242 was used to verify the involvement of TLR4 signaling.

**Results:**

In vitro, zonisamide suppressed morphine-induced upregulation of interleukin (IL)-1β, IL-6, tumor necrosis factor (TNF)-α, and Toll-like receptor 4 (TLR4) mRNA, reduced morphine-induced secretion of IL-1β, IL-6, and TNF-α at the protein level, and inhibited morphine-induced p38 mitogen-activated protein kinase (MAPK) phosphorylation. In vivo, zonisamide did not affect acute morphine analgesia but significantly attenuated the development of chronic morphine tolerance. Spinal cord analyses revealed that zonisamide reduced microglial activation (Iba1), TLR4 expression, p38 phosphorylation, and pro-inflammatory cytokine (IL-1β, TNF-α) protein levels. Pharmacological inhibition of TLR4 with TAK-242 produced a behavioral effect similar to that of zonisamide, and combined treatment did not provide additional benefit, suggesting overlapping signaling effects.

**Discussion:**

These findings indicate that zonisamide attenuates morphine tolerance in association with reduced spinal TLR4/p38 MAPK signaling and neuroinflammation. This study provides a mechanistic rationale for repurposing zonisamide as an adjunct therapy to improve long-term pain management and mitigate opioid-related adverse effects.

## 1 Introduction

Morphine remains a cornerstone in the management of moderate to severe pain, yet its long-term clinical utility is significantly compromised by the development of analgesic tolerance. In a 2-year prospective cohort of 517 patients receiving stable long-term opioid therapy for musculoskeletal pain, 19.5% underwent dose escalation, but escalation was not associated with improved pain outcomes (Morasco et al., 2020). At the mechanistic level, opioid-induced analgesic tolerance can necessitate dose escalation and thereby increase exposure to opioid-related adverse effects (Caraceni et al., 2012; Christie, 2008). A growing body of preclinical evidence implicates neuroinflammatory signaling, including microglial reactivity and the release of interleukin-1β (IL-1β), interleukin-6 (IL-6), and tumor necrosis factor-α (TNF-α), in the development of morphine tolerance (Zhang et al., 2017; Gei et al., 2024). Therefore, identifying pharmacological agents that can suppress this neuroinflammatory response without compromising morphine’s acute analgesic effect represents a promising therapeutic strategy.

Current research has established that morphine directly engages microglial cells, in part through Toll-like receptor 4 (TLR4), a pattern recognition receptor traditionally associated with pathogen recognition (Wang et al., 2021; Pan et al., 2016; Qu et al., 2017). This interaction triggers intracellular signaling cascades, most notably the p38 mitogen-activated protein kinase (MAPK) pathway, leading to the transcriptional upregulation of pro-inflammatory cytokines (Jiang et al., 2014; Cui et al., 2007). The resulting neuroinflammatory milieu in pain-processing regions, such as the spinal cord dorsal horn, is now recognized as a key mechanism that drives the transition from acute analgesia to chronic tolerance. Despite this mechanistic understanding, effective pharmacological interventions to prevent or reverse morphine tolerance remain scarce. While several anti-inflammatory compounds have shown preclinical promise, many lack clinical approval or are associated with significant side effects, highlighting a critical gap in the development of safe, repurposable therapies.

Zonisamide, an antiepileptic drug approved for the treatment of partial seizures, has recently garnered attention for its pleiotropic effects beyond neuronal stabilization (Hoy, 2013). Preclinical studies have demonstrated that zonisamide possesses potent anti-inflammatory and neuroprotective properties in various CNS pathologies, including cerebral ischemia (Minato et al., 1997) and neurodegenerative diseases (Asanuma et al., 2010; Supuran, 2024). These effects are mediated, in part, through the suppression of microglial activation and the inhibition of pro-inflammatory cytokine release (Koshimizu et al., 2020; Hossain et al., 2018). However, the potential of zonisamide to specifically counteract morphine-induced neuroinflammation and its downstream consequence—analgesic tolerance—has not been investigated. This represents a significant knowledge gap, as a clinically approved drug with a known safety profile could be rapidly repurposed to address this major limitation of opioid therapy.

To address this gap, the present study investigated whether zonisamide attenuates morphine-induced neuroinflammation and analgesic tolerance through the suppression of microglial activation and the TLR4/p38 MAPK signaling pathway. The specific objectives were threefold: 1) to determine whether zonisamide inhibits morphine-induced pro-inflammatory cytokine expression and TLR4/p38 MAPK signaling in BV-2 microglial cells; 2) to evaluate the effect of zonisamide on the development of morphine tolerance in C57BL/6 mice, while confirming that it does not interfere with acute morphine analgesia; and 3) to investigate whether zonisamide’s behavioral effects are associated with a reduction in microglial activation and the suppression of the TLR4/p38 MAPK pathway in the spinal cord. By elucidating these mechanisms, this study provides the first evidence for zonisamide as a potential adjunctive therapy to preserve morphine’s analgesic efficacy, thereby addressing a critical unmet need in pain management.

## 2 Results

### 2.1 Zonisamide inhibits morphine-induced inflammation in BV-2 microglial cells

**FIGURE 1:**
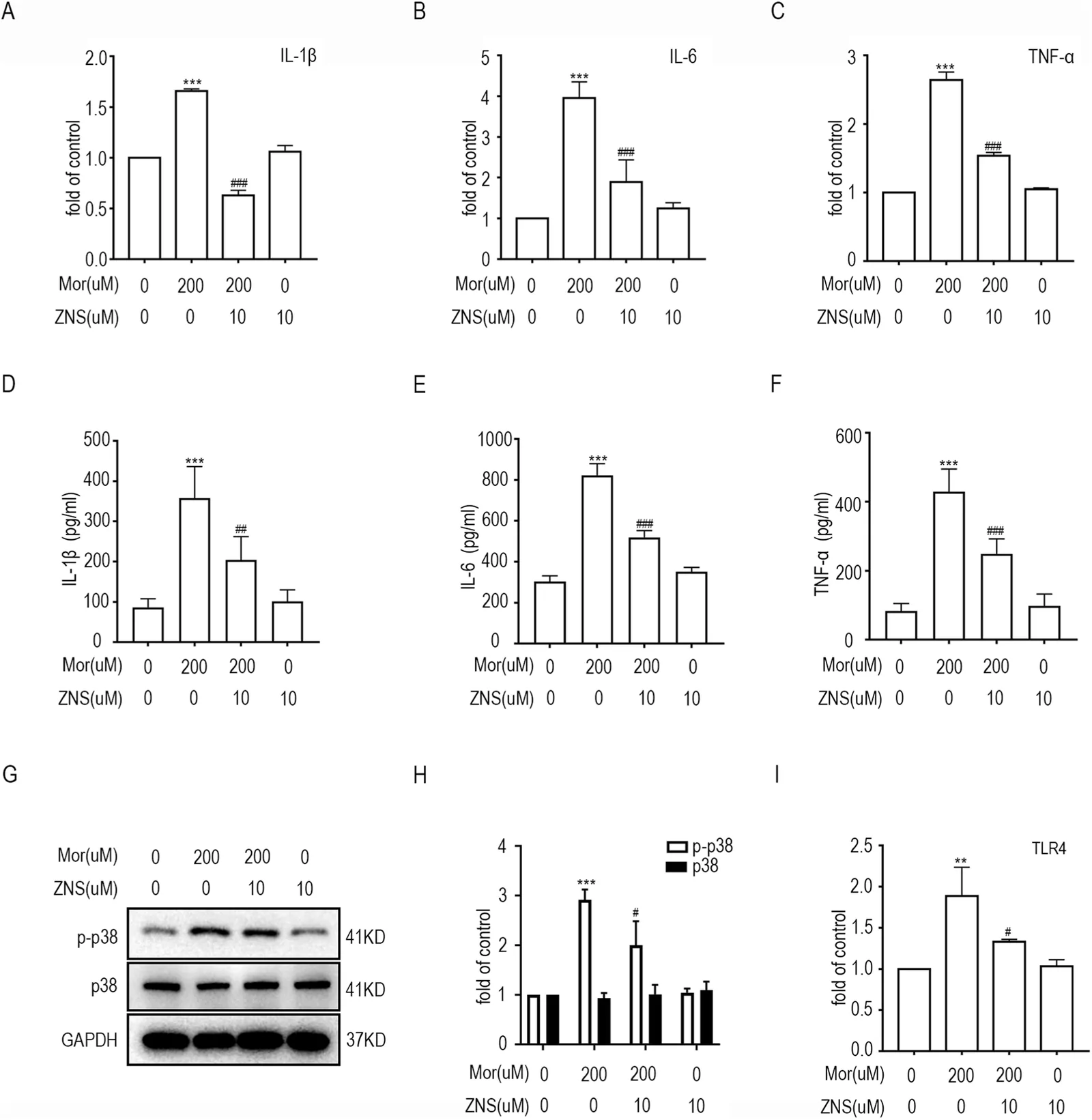
Zonisamide inhibits morphine-triggered inflammation in BV-2 microglial cells. **(A–C)** Zonisamide restricted the upregulation of pro-inflammatory cytokine mRNA induced by morphine in BV-2 cells. The mRNA levels of IL-1β **(A)**, IL-6 **(B)**, and TNF-α **(C)** were measured by real-time quantitative PCR and normalized to GAPDH (n = 3). **(D–F)** Zonisamide reduced the protein levels of IL-1β **(D)**, IL-6 **(E)**, and TNF-α **(F)** induced by morphine in BV-2 cells, as determined by ELISA (n = 4). **(G,H)** Zonisamide impeded morphine-induced p38 phosphorylation in BV-2 cells. (n = 4). **(I)** Zonisamide repressed the expression of TLR-4 mRNA induced by morphine in BV-2 cells, measured by real-time quantitative PCR (n = 3). Data are presented as mean ± SD. Statistical analysis was performed using one-way ANOVA followed by Bonferroni *post hoc* test (A: F(3,8) = 320.9, *P* = 1.13 × 10^−8^; **(B)** F(3,8) = 45.86, *P* = 2.19 × 10^−5^; **(C)** F(3,8) = 431.2, *P* = 3.50 × 10^−9^; **(D)** F(3,12) = 23.14, *P* = 2.80 × 10^−5^; **(E)** F(3,12) = 157.8, *P* = 6.63 × 10^−10^; **(F)** F(3,12) = 53.08, *P* = 3.37 × 10^−7^; **(H)** F(3,12) = 37.39, *P* = 2.29 × 10^−6^; **(I)** F(3,8) = 15.81, *P* = 1.00 × 10^−3^). **P < 0.01 and ***P < 0.001 versus the saline group; #*P* < 0.05, ##*P* < 0.01, and ###*P* < 0.001 versus the morphine-treated group. Abbreviations: Mor, morphine; ZNS, zonisamide.

To investigate the anti-inflammatory effect of zonisamide on morphine-stimulated microglia, BV-2 cells were treated with morphine (200 μM) (Pan et al., 2016) in the presence or absence of zonisamide (10 μM) (Koshimizu et al., 2020) for 12 h. The concentrations were selected based on previous studies and were confirmed to be non-cytotoxic by CCK-8 assay, which showed no significant difference in cell viability among treatment groups (Supplementary Figure S1). Morphine exposure significantly upregulated the mRNA levels of IL-1β, IL-6, and TNF-α, as determined by real-time quantitative PCR. Zonisamide co-treatment markedly suppressed the elevated mRNA expression levels of these pro-inflammatory cytokines (Figures 1A–C). Consistently, ELISA analysis revealed that zonisamide also significantly reduced morphine-induced protein levels of these pro-inflammatory cytokines (Figures 1D–F), confirming that the anti-inflammatory effect of zonisamide occurs at both the transcriptional and translational levels. Furthermore, morphine upregulated TLR4 mRNA expression and increased p38 MAPK phosphorylation, both of which were significantly attenuated by zonisamide co-treatment (Figures 1G–I). These results indicate that zonisamide suppresses morphine-induced microglial activation through inhibition of the TLR4/p38 MAPK signaling pathway.

### 2.2 Zonisamide attenuates chronic morphine tolerance in mice

**FIGURE 2:**
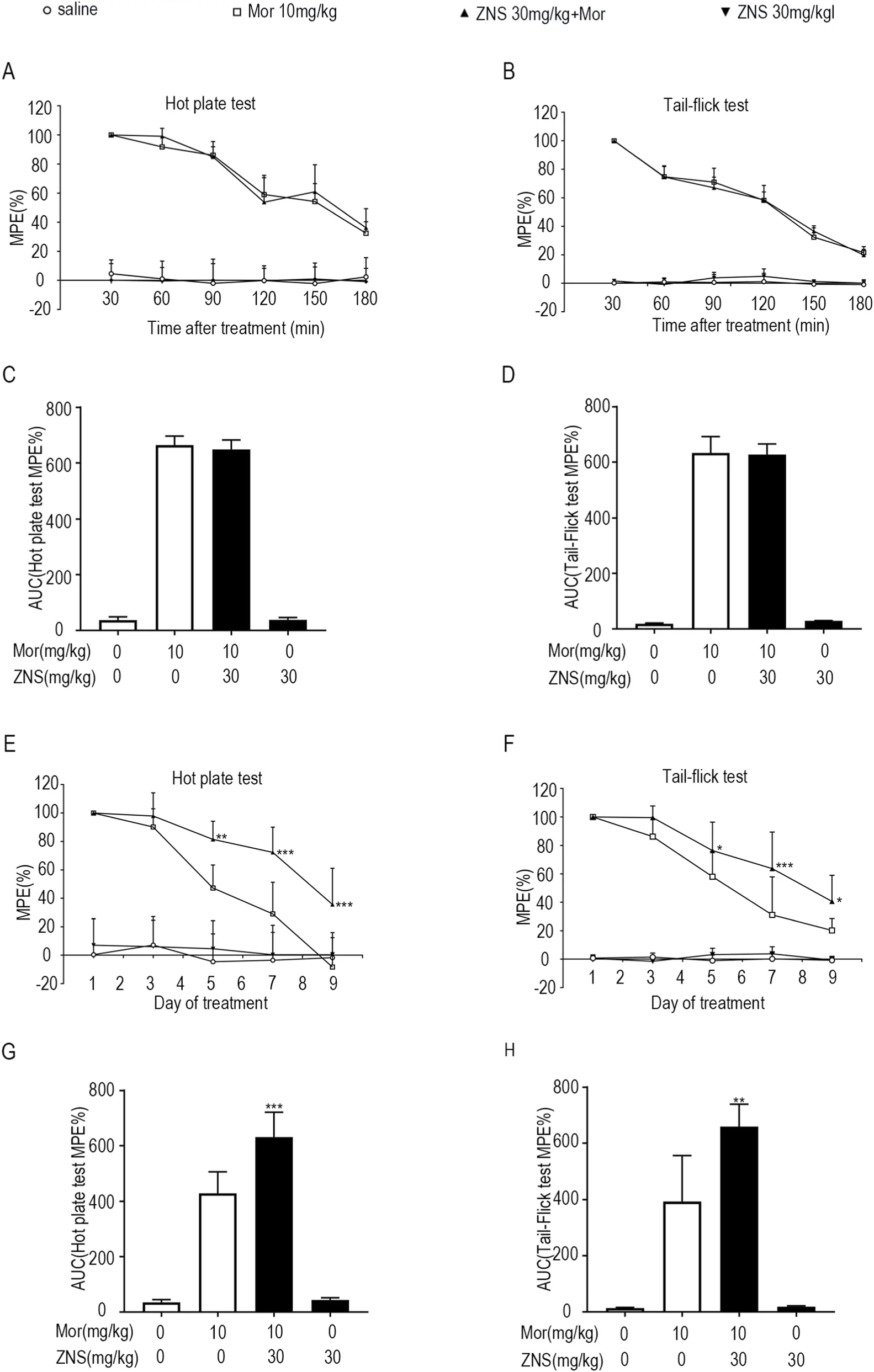
Zonisamide mitigated the development of chronic morphine tolerance. **(A,B)** Time course of the antinociceptive response following a single acute administration. Mice received a single injection of morphine (10 mg/kg, s.c.) with or without zonisamide (30 mg/kg, i.p.). Nociceptive thresholds were measured at 30, 60, 90, 120, 150, and 180 min post-injection using the hot-plate test **(A)** and tail-flick test **(B)**. Data are presented as mean ± SD (n = 6 mice per group). Two-way repeated measures ANOVA revealed a significant treatment × time interaction (**(A)** F(15,75) = 10.4, *P* = 6.87 × 10^−13^; **(B)** F(15,75) = 72.13, *P* = 2.2 × 10^−34^). Bonferroni *post hoc* comparisons showed no significant differences between the morphine alone and morphine + zonisamide groups at any time point (*P* > 0.05). **(C,D)** Area under the curve (AUC) analysis of the antinociceptive response over the 180-min observation period. One-way ANOVA with Bonferroni *post hoc* test confirmed no significant difference between the morphine alone and morphine + zonisamide groups (**(C)** F(3,20) = 1,249, *P* = 2.74 × 10^−29^; **(D)** F(3,20) = 591.4, *P* = 1.19 × 10^−19^). **(E,F)** Time course of the antinociceptive response over nine consecutive days of repeated morphine administration. Mice received morphine (10 mg/kg, s.c., twice daily) with or without zonisamide (30 mg/kg, i.p.). The hot - plate test **(E)** and the tail - flick test **(F)** were performed on days 1, 3, 5, 7, and 9. Data are presented as mean ± SD (n = 6 mice per group). Two-way repeated measures ANOVA revealed a significant treatment × time interaction (**(E)** F(12,60) = 9.975, *P* = 2.36 × 10^−10^; **(F)** F(12,60) = 14.42, *P* = 1.62 × 10^−13^). Bonferroni *post hoc* comparisons showed that the morphine + zonisamide group exhibited significantly higher nociceptive thresholds compared to the morphine alone group at multiple time points. *P < 0.05, **P < 0.01, ***P < 0.001 versus the morphine group. **(G,H)** Area under the curve (AUC) analysis of the antinociceptive response over the 9-day treatment period. One-way ANOVA followed by Bonferroni *post hoc* test (**(G)** F(3,20) = 147.2, *P* = 8.4 × 10^−14^; **(H)** F(3,20) = 71.1, *P* = 7.62 × 10^−11^). **P < 0.01, ***P < 0.001 versus versus the morphine group. Abbreviations: Mor, morphine; ZNS, zonisamide.

To evaluate whether zonisamide prevents the development of chronic morphine tolerance, mice received twice-daily injections of morphine (10 mg/kg, s.c.) with or without zonisamide for nine consecutive days. In the morphine-only group, the antinociceptive effect progressively declined over the treatment period in both the hot-plate and tail-flick tests, indicating the development of analgesic tolerance. Co-administration of zonisamide significantly attenuated this decline (Figures 2E,F). AUC analysis further confirmed that the zonisamide-treated group maintained significantly greater antinociception compared with the morphine-only group (Figures 2G,H).

To determine whether zonisamide interferes with the acute antinociceptive effect of morphine, mice received a single injection of morphine (10 mg/kg, s.c.) with or without zonisamide pretreatment (30 mg/kg, i.p.). As shown in Figures 2A–D, zonisamide did not alter the peak antinociceptive effect of morphine or the area under the curve (AUC) in either the hot-plate or tail-flick test, indicating that zonisamide does not compromise acute opioid analgesia. No significant differences in baseline nociceptive thresholds were observed among the groups during the 9-day treatment period (Supplementary Figure S2).

Pharmacological inhibition of TLR4 with TAK-242 (3 mg/kg, i.p.) partially recapitulated the protective effect of zonisamide, and no additional benefit was observed when zonisamide was combined with TAK-242 (Supplementary Figure S3), suggesting that zonisamide’s anti-tolerance effect is at least partially dependent on TLR4 signaling.

### 2.3 Zonisamide suppresses spinal microglial activation and TLR4/p38 MAPK signaling in mice with morphine tolerance

**FIGURE 3:**
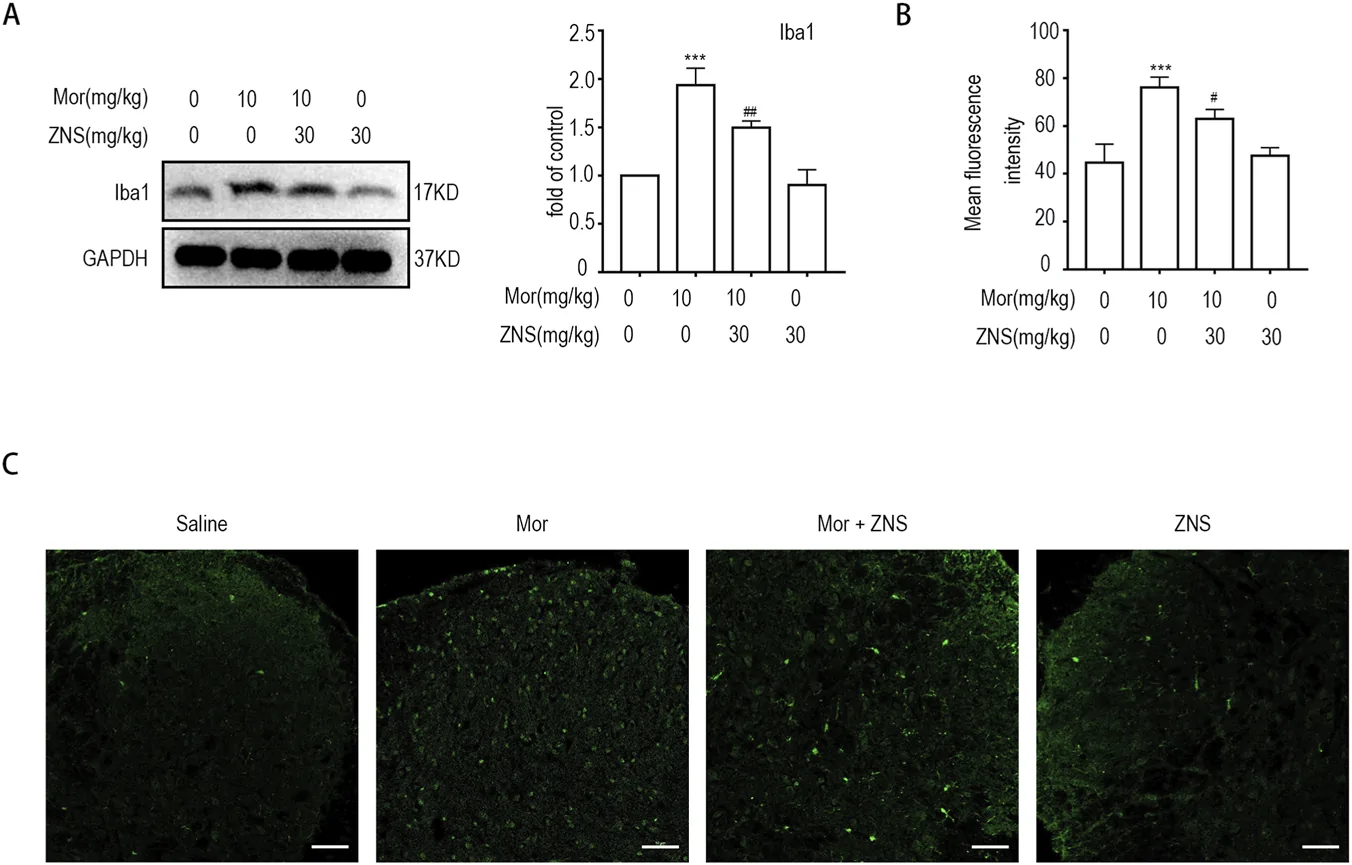
Zonisamide attenuated spinal microglial activation in chronic morphine administration. **(A)** Zonisamide suppressed the upregulation of Iba1 protein expression in the spinal cord following chronic morphine treatment, as determined by Western blot analysis (n = 4). **(B,C)** Zonisamide inhibited the expression of Iba1 induced by long-term morphine administration, as assessed by confocal immunofluorescence imaging (n = 3). Representative confocal images and quantitative analysis of Iba1 immunoreactivity in the dorsal horn of the spinal cord are shown (scale bar, 50 μm). Data are presented as mean ± SD. Statistical analysis was performed using one-way ANOVA followed by Bonferroni *post hoc* test (**(A)** F(3, 12) = 45.95, *P* = 7.47 × 10^−7^; **(B)** F(3, 8) = 27.14, *P* = 1.52 × 10^−4^). ***P < 0.001 versus the Saline group; #*P* < 0.05 and ##*P* < 0.01 versus the morphine-treated group. Abbreviations: Mor, morphine; ZNS, zonisamide.

To investigate whether zonisamide’s behavioral effects are associated with reduced microglial activation, we examined Iba1 expression in the spinal cord dorsal horn. Morphine administration significantly increased Iba1 protein expression, as determined by Western blot analysis, and this upregulation was markedly suppressed by zonisamide co-treatment (Figure 3A). Immunofluorescence staining further confirmed that zonisamide reduced morphine-induced Iba1 immunoreactivity in the dorsal horn (Figures 3B,C).

**FIGURE 4:**
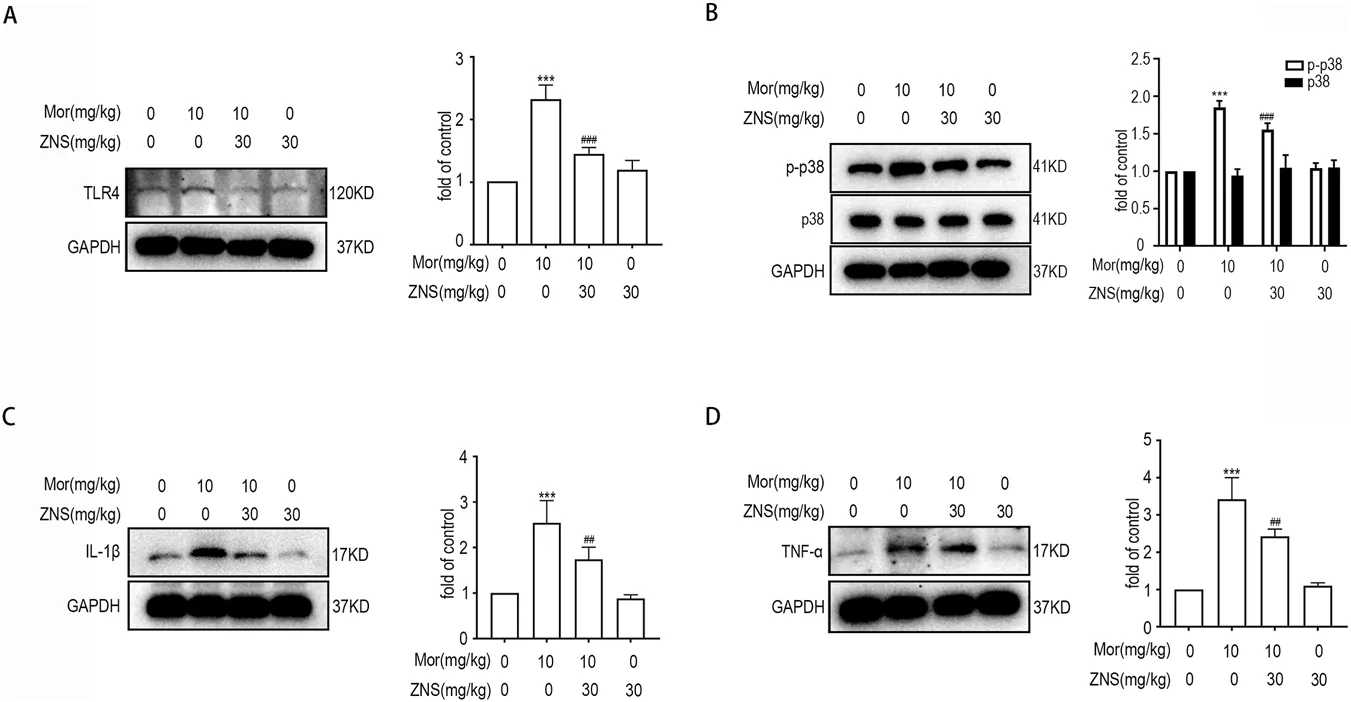
Zonisamide suppresses morphine-induced inflammation in the spinal cord *in vivo*. **(A)** Zonisamide inhibited the upregulation of TLR-4 protein expression in the spinal cord induced by chronic morphine treatment (n = 4). **(B)** Zonisamide suppressed morphine-induced p38 phosphorylation in the spinal cord (n = 4). **(C,D)** Zonisamide reduced the protein expression of IL-1β **(C)** and TNF-α **(D)** in the spinal cord following chronic morphine administration (n = 4). Data are presented as mean ± SD. Statistical analysis was performed using one-way ANOVA followed by Bonferroni *post hoc* test (**(A)** F(3,12) = 59, *P* = 1.87 × 10^−7^; **(B)** F(3,12) = 139.5, *P* = 1.36 × 10^−9^; **(C)** F(3,12) = 30.06, *P* = 7.29 × 10^−6^; **(D)** F(3,12) = 56.08, *P* = 2.48 × 10^−7^). ***P < 0.001 versus the Saline group; ##P < 0.01 and ###P < 0.001 versus the morphine-treated group. Abbreviations: Mor, morphine; ZNS, zonisamide.

To further elucidate the molecular mechanisms underlying zonisamide’s anti-tolerance effect, we measured the expression of TLR4, phosphorylated p38 (p-p38), and pro-inflammatory cytokines in the spinal cord dorsal horn. Morphine treatment significantly upregulated TLR4 mRNA expression and increased the p-p38, both of which were significantly attenuated by zonisamide co-treatment (Figures 4A,B). In addition, morphine-induced increases in the protein levels of IL-1β and TNF-α were significantly reduced by zonisamide (Figures 4C,D). These findings demonstrate that zonisamide suppresses morphine-induced neuroinflammation in the spinal cord through inhibition of the TLR4/p38 MAPK signaling cascade.

## 3 Discussion

Morphine remains a cornerstone in the management of moderate to severe pain, yet its long-term clinical utility is severely compromised by the development of analgesic tolerance, a phenomenon characterized by a progressive decline in drug efficacy requiring dose escalation (Williams et al., 2013). This limitation not only diminishes pain control but also increases the risk of adverse effects and opioid misuse. Emerging evidence has established that morphine exposure induces a state of neuroinflammation, primarily driven by the activation of microglial cells within the central nervous system (Zhang et al., 2024; Wen et al., 2011). This neuroimmune response, mediated through pathways such as Toll-like receptor 4 (TLR4) and p38 MAPK, leads to the release of pro-inflammatory cytokines like IL-1β, IL-6, and TNF-α, which are now recognized as critical contributors to the pathogenesis of opioid tolerance (Li et al., 2020; Cao et al., 2025; Wang et al., 2022; Chen and Sommer, 2009). Consequently, identifying pharmacological agents that can safely mitigate this neuroinflammatory cascade without compromising morphine’s acute analgesic effect represents a significant therapeutic opportunity.

The present study demonstrates that zonisamide, a widely used antiepileptic agent with known anti-inflammatory properties, effectively attenuates the development of chronic morphine tolerance without compromising acute analgesic efficacy. Importantly, this protective effect was associated with reduced TLR4/p38 MAPK signaling and decreased neuroinflammation. These findings provide a preclinical rationale for future clinical evaluation of zonisamide as an adjunct therapy in long-term opioid management.

Morphine exposure is known to induce a state of neuroinflammation characterized by the activation of spinal microglia and the upregulation of pro-inflammatory cytokines such as IL-1β, IL-6, and TNF-α (Liu et al., 2019; Tian et al., 2025; Xie et al., 2024). In agreement with these previous observations, our *in vitro* data showed that morphine treatment significantly increased the mRNA levels of these cytokines in BV-2 microglial cells, along with TLR4 expression. Zonisamide co-treatment effectively reversed these changes, indicating that zonisamide can directly counteract morphine-induced microglial activation at the transcriptional level. Furthermore, Western blot analysis revealed that zonisamide blocked morphine-induced phosphorylation of p38 MAPK, a key downstream effector of TLR4 signaling that is critically involved in the synthesis of inflammatory mediators (Han et al., 2014). These findings suggest that the TLR4/p38 axis may be a key target associated with zonisamide’s anti-neuroinflammatory effects.

Our *in vivo* results corroborated these *in vitro* findings. Repeated systemic administration of morphine led to a progressive decline in nociceptive thresholds in both hot plate and tail flick tests, confirming the establishment of analgesic tolerance. Zonisamide treatment significantly slowed this tolerance development, yet it did not alter the acute antinociceptive response to morphine. This dissociation is clinically important, as it implies that zonisamide may prolong the utility of opioids without diminishing the rapid pain relief required for breakthrough episodes. To exclude the possibility that baseline differences in pain sensitivity confounded these results, we measured baseline nociceptive thresholds throughout the treatment period; no significant differences were observed among the experimental groups, confirming that the behavioral changes reflect genuine alterations in tolerance rather than pre-existing disparities in pain sensitivity (Supplementary Figure S2).To further address whether the protective effect of zonisamide is causally dependent on TLR4 signaling, we employed a pharmacological TLR4 inhibitor (TAK-242). Administration of TAK-242 partially recapitulated the protective effect of zonisamide, and the combination of zonisamide and TAK-242 did not produce additional benefit beyond either treatment alone (Supplementary Figure S3). These results suggest that zonisamide and TAK-242 act on a shared signaling axis—the TLR4 pathway—supporting the interpretation that zonisamide’s anti-tolerance effect may involve the inhibition of TLR4-dependent neuroinflammation. However, the lack of a statistically significant difference among the zonisamide, TAK-242, and combination groups precludes a definitive conclusion regarding complete mechanistic occlusion. Future studies employing TLR4-deficient mice or genetic knockdown approaches would provide stronger causal evidence.

At the spinal level, zonisamide treatment reduced the expression of Iba1 (a marker of microglial activation), TLR4, and phospho-p38, and decreased the protein levels of IL-1β and TNF-α. These histological and biochemical data collectively confirm that zonisamide mitigates the neuroinflammatory milieu in the spinal cord that underlies tolerance. The involvement of TLR4 in opioid tolerance has been extensively documented. Morphine can directly bind to TLR4 on microglia, triggering a non-classical, non-opioid receptor-mediated inflammatory cascade that contributes to tolerance and hyperalgesia (Wang et al., 2020; Ahmadi et al., 2025). By inhibiting TLR4 expression and downstream p38 phosphorylation, zonisamide appears to disrupt this cascade. Notably, zonisamide is already known to possess anti-inflammatory and neuroprotective properties in other central nervous system disorders, such as epilepsy and Parkinson’s disease (Kumar et al., 2018; Aşır et al., 2024). The present study extends these properties to the context of opioid-induced neuroinflammation, suggesting a novel therapeutic application for this established drug.

From a translational perspective, the potential repurposing zonisamide offers several advantages. A pilot clinical study has demonstrated the safety and tolerability of zonisamide in patients with painful diabetic neuropathy (Atli and Dogra, 2005), supporting its feasibility in pain management. As an approved medication with a well-characterized safety profile, its clinical use for preventing morphine tolerance could be rapidly implemented (Kothare and Kaleyias, 2008). The fact that zonisamide does not interfere with morphine’s acute analgesic effect is particularly appealing, as it avoids the risk of immediate pain breakthrough. Moreover, given that neuroinflammation is also implicated in other opioid-related adverse effects, such as opioid-induced hyperalgesia and reward sensitization, zonisamide may have broader benefits beyond tolerance. Nevertheless, several limitations of this study should be acknowledged. First, the *in vitro* experiments were conducted exclusively in the BV-2 microglial cell line, which may not fully recapitulate the responses of primary microglia. Future validation in primary mouse microglia is warranted. Second, only a single dose of zonisamide was tested, and experiments were conducted exclusively in male mice; dose-response studies and investigations in female animals are necessary to determine the optimal therapeutic window and assess sex-dependent effects. Third, although we focused on microglial TLR4/p38 signaling, zonisamide may also modulate additional signaling cascades (e.g., NF-κB or NLRP3 inflammasome), and the lack of cell-type-specific evidence, such as double immunofluorescence staining of Iba1 with TLR4 or p-p38, limits the confirmation that this pathway is specifically activated in spinal microglia. Moreover, preliminary GFAP staining suggested a potential effect on astrocytes (Supplementary Figure S4); future studies are warranted to systematically investigate the role of astrocytes in zonisamide’s anti-tolerance effects. Finally, formal behavioral assessments of locomotor activity and motor coordination were not performed due to equipment limitations; however, no overt signs of sedation or motor impairment were observed in zonisamide-treated animals. The long-term effects of combined zonisamide and morphine treatment on cognitive function and physical dependence also remain to be explored.

In conclusion, our findings indicate that zonisamide attenuates morphine tolerance in association with reduced spinal TLR4/p38 MAPK signaling and neuroinflammation. This study provides a preclinical rationale for the potential repurposing of zonisamide as an adjunct therapy to improve the long-term management of pain and reduce the limitations associated with opioid tolerance.

## 4 Materials and methods

### 4.1 Animals

Adult male C57BL/6 mice (18–20 g) were obtained from the Guangdong Medical Laboratory Animal Center (Guangzhou, China). All animal experiments were approved by the Animal Care and Use Committee of the Sun Yat-sen University Cancer Center (approval No. L025501202306003). Mice were quarantined for 7 days prior to experiments and housed in groups of up to five per cage under a constant temperature with a 12-h light/dark cycle and *ad libitum* access to food and water. All efforts were made to minimize the number of animals used and their suffering.

### 4.2 Chemicals and reagents

Morphine hydrochloride was purchased from Shenyang First Pharmaceutical Factory, Northeast Pharmaceutical Group Company (Shenyang, China). Zonisamide (HY-B0124) and TAK-242 (HY-11109) was purchased from MedChemExpress (MCE, Monmouth Junction, NJ, United States). Antibodies against GAPDH (60004-1-Ig) were purchased from ProteinTech Group (Chicago, IL, United States). Antibodies against p38 (49379) and phosphorylated p38 (Tyr182) (11253) were purchased from Signalway Antibody (Nanjing, China). Antibodies against IL-1β (AF5103) and TNF-α (AF7014) were purchased from Affinity (Cincinnati, United States). Antibodies against Iba1 (ab5076) and TLR4 (ab13556) were purchased from Abcam (Cambridge, MA, United States). Secondary antibodies for Western blotting were from Sigma‒Aldrich (St. Louis, MO, United States). Immunofluorescent antibodies against Iba1 (ab5076) was purchased from Abcam (Cambridge, MA, United States). Antibodies against GFAP antibody (3670T) was purchased from Cell Signaling Technology (Danvers, MA, United States). Secondary antibodies for immunofluorescence were from Jackson ImmunoResearch Laboratories (West Grove, PA, United States). Fetal bovine serum (FBS), other cell culture media, and supplements were purchased from Gibco (Thermo Fisher Scientific, Waltham, MA, United States).

### 4.3 Induction of morphine tolerance and behavioral tests

Chronic morphine tolerance was induced as previously described (Trujillo and Akil, 1991; Jouvenel et al., 2024). Mice received subcutaneous injections of morphine (10 mg/kg) or an equal volume of saline twice daily (at 08:00 and 18:00) for nine consecutive days. Nociceptive testing was performed 30 min after morphine administration on days 1, 3, 5, 7, and 9. All behavioral tests were conducted by an experimenter blinded to group assignments.

#### 4.3.1 Tail - flick test

Thirty minutes after morphine treatment, the tail-flick test was conducted following established protocols (Zhang et al., 2017). The distal two-thirds of the mouse’s tail was immersed in a 52 °C water bath, and the latency to tail withdrawal was recorded. A maximum cutoff time of 10 s was set to prevent tissue damage. Data were expressed as a percentage of the maximal possible effect (%MPE), calculated using the formula: 100% × [(Drug response time − Basal response time)/(10 s − Basal response time)] = %MPE.

#### 4.3.2 Hot plate test

On odd days, 30 min after morphine administration, the hot plate test was carried out as described previously (Abdel et al., 2010). Mice were placed on a 55 °C hot-plate surface within a plexiglass cylinder, and the latency to hind-paw licking or jumping was recorded as the endpoint. A maximum cutoff time of 30 s was applied to prevent tissue damage. Mice were habituated to the apparatus for 3 days prior to the experiment. Data were calculated as a percentage of the maximal possible effect (%MPE) using the formula: 100% × [(Drug response time − Basal response time)/(30 s − Basal response time)] = %MPE.

##### 4.3.2.1 Acute analgesic effect of morphine

To assess whether zonisamide affects the acute analgesic effect of morphine, a separate cohort of mice received a single injection of morphine (10 mg/kg, s.c.) with or without zonisamide (30 mg/kg, i.p.). Nociceptive responses were measured using the hot-plate and tail-flick tests at 30, 60, 90, 120, 150, and 180 min after morphine administration. Data were expressed as percentage of maximal possible effect (%MPE).

##### 4.3.2.2 Pharmacological inhibition of TLR4

To investigate the role of TLR4 signaling in morphine tolerance, mice were randomly assigned to the following groups: (1) Vehicle + Saline, (2) Vehicle + Morphine, (3) Zonisamide + Morphine, (4) TAK-242 + Morphine, and (5) Zonisamide + TAK-242 + Morphine. TAK-242 (3 mg/kg, i.p.) or vehicle was administered 30 min prior to each morphine injection. Morphine tolerance was induced as described above. Behavioral tests were performed on days 1, 3, 5, 7, and 9.

### 4.4 Cell cultures

BV-2 mouse microglial cells were cultured in Dulbecco’s modified Eagle’s medium (DMEM) supplemented with 10% (v/v) FBS, 100 U/mL penicillin, and 100 μg/mL streptomycin in a humidified atmosphere containing 5% CO_2_ at 37 °C. For experiments, 1 × 10^5^ cells were seeded in 6-well or 12-well plates and allowed to adhere overnight. The following day, cells were treated with morphine (200 μM) in the presence or absence of zonisamide (10 μM) for 12 h. Cell lysates and supernatants were collected for subsequent analysis by immunoblotting or real-time PCR.

### 4.5 Cell viability assay

Cell viability was assessed using the Cell Counting Kit-8 (GOONIE, Shanghai, China) according to the manufacturer’s instructions. Briefly, BV-2 cells were seeded in 96-well plates and treated with morphine (200 μM) and/or zonisamide (10 μM) for 12 h. CCK-8 reagent was added to each well and incubated for 2 h at 37 °C. Absorbance was measured at 450 nm using a microplate reader. Cell viability was expressed as a percentage relative to the control group.

### 4.6 Enzyme-linked immunosorbent assay (ELISA)

BV-2 cells were treated as described above, and cell culture supernatants were collected. Protein levels of IL-1β, TNF-α, and IL-6 were measured using commercial ELISA kits according to the manufacturers’ instructions. The following kits were used: mouse IL-1β ELISA kit (KE10003, Proteintech, Rosemont, IL, United States), mouse TNF-α ELISA kit (RK00027, ABclonal, Wuhan, China), and mouse IL-6 ELISA kit (PK00008, ABclonal, Wuhan, China). Absorbance was read at 450 nm using a microplate reader.

### 4.7 Quantitative RT‒PCR

Spinal cord tissues (L4–L5 segments) were collected and rapidly frozen on dry ice. Under a dissecting microscope, the spinal cord was cut transversely at the midline of the central canal using a microsurgical blade. The ventral horn (anterior two-thirds) was carefully dissected and discarded, leaving the dorsal horn (posterior one-third) for RNA extraction. Total RNA was then extracted from the dorsal horn using a Tissue RNA Purification Kit Plus (ESscience, Shanghai, China) according to the manufacturer’s instructions. For BV-2 cells, total RNA was extracted using TRIzol reagent (Invitrogen, Carlsbad, CA, United States). cDNA was synthesized using an EZBioscience Color Reverse Transcription Kit (Roseville, MN, United States). Real-time quantitative PCR was performed using SYBR Green (EZBioscience, Roseville, MN, United States) on a CFX96 Touch Real-Time PCR System (Bio-Rad, Hercules, CA, United States). The following primer sequences were used: GAPDH forward: CATCACTGCCACCCAGAAGACTG, reverse: ATGCCAGTGAGCTTCCCGTTCAG; TLR4 forward: GCCATCATTATGAGTGCCAAT, reverse: AGGGATAAGAACGCTGAGAATT; IL-6 forward: AGACAGCCACTCACCTCTTCAG, reverse: TTCTGCCAGTGCCTCTTTGCTG; IL-1β forward: TGGACCTTCCAGGATGAGGACA, reverse: GTTCATCTCGGAGCCTGTAGTG; TNF-α forward: GTGTGGCTGTAAGGAGAACCAG, reverse: CACACGGTGTTCTGAGTCTCCT. Each sample was run in technical triplicate, and the mean Ct value was used for analysis. Target mRNA expression was normalized to GAPDH using the 2^−ΔΔCt method. Three independent biological replicates were performed for each experiment.

### 4.8 Western blotting

Cells or spinal dorsal horn tissues (L4–L5 segments) were collected and stored at −80 °C until analysis. Samples were lysed in RIPA buffer containing 1% NP-40, 0.5% sodium deoxycholate, 0.1% SDS, and protease inhibitors. After centrifugation at 12,000 × g for 5 min, the supernatant was collected. Protein samples were separated by 10% SDS-PAGE and electrotransferred onto polyvinylidene fluoride (PVDF) membranes (Millipore, Billerica, MA, United States). Membranes were blocked with 10% non-fat milk in TBST (Tris-HCl, NaCl, 0.1% Tween 20) for 1 h at room temperature and then incubated overnight at 4 °C with primary antibodies against IL-1β (1:1,000), IL-α (1:1,000), TLR4 (1:1,000), Iba1 (1:1,000), p-p38 (Tyr182) (1:1,000), p38 (1:1,000), and GAPDH (1:10,000). The following day, membranes were incubated with horseradish peroxidase (HRP)-conjugated secondary antibodies for 1 h at room temperature. Protein bands were visualized using enhanced chemiluminescence reagents (Millipore) and imaged with a Molecular Imager and Quantity One software (Bio-Rad, Hercules, CA, United States). Densitometric analysis was performed using ImageJ software. The expression levels of phospho-p38 were normalized to total p38, and the expression levels of TLR4, IL-1β, and TNF-α were normalized to GAPDH.

### 4.9 Immunofluorescence

Mice were put under anesthesia through an intraperitoneal injection of sodium pentobarbital (100 mg/kg). Subsequently, they underwent transcardial perfusion. First, phosphate - buffered saline (PBS) was used for perfusion, followed by 4% paraformaldehyde. The L4 – L5 segments of the mice were then excised and immersed in 4% paraformaldehyde for 3 h. After that, they were transferred to a 30% sucrose solution at 4 °C and left there until the tissue settled at the bottom of the container. Transverse spinal cord sections with a thickness of 25 μm were cut using a cryostat. The subsequent steps were carried out as previously described (Chu et al., 2020). The sections were first treated with Immunol Staining Blocking Buffer (Beyotime, China) for 1 h at room temperature. Then, they were incubated with primary antibodies mouse monoclonal anti - Iba1 (1:200) or anti-GFAP (1:100). This incubation lasted for 24 h at 4 °C. After being washed three times with PBS, the sections was further incubated with FITC - Alexa Fluor 488 secondary antibodies (1:500) for 1 h at room temperature. All images were captured using a confocal microscope (LSM980, ZEISS, Germany). The data obtained were analyzed with ImageJ software.

### 4.10 Data analysis and statistics

Data are presented as mean ± SD from independent biological replicates (n per group as indicated in the figure legends). Statistical analyses were performed using GraphPad Prism version 9 (GraphPad Software, San Diego, CA, United States). For comparisons among multiple groups, one-way analysis of variance (ANOVA) followed by Bonferroni’s *post hoc* test was used. For behavioral data with repeated measurements over time, two-way repeated-measures ANOVA followed by Bonferroni’s *post hoc* test was applied. A P value less than 0.05 was considered statistically significant.

## Data Availability

The original contributions presented in the study are included in the article/Supplementary Material, further inquiries can be directed to the corresponding authors.
