# Supplementary material for: Zonisamide attenuates morphine tolerance in association with reduced spinal TLR4/p38 MAPK signaling
Source: Front Pharmacol. 2026 Aug 26;17:1893236. doi: 10.3389/fphar.2026.1893236 (PMC13553760; doi:10.3389/fphar.2026.1893236)
Supplement: Supplementary file 1 [file Supplementaryfile1.docx]

Supplementary Materials for

**Zonisamide Attenuates Morphine Tolerance in Association with Reduced Spinal TLR4/p38 MAPK Signaling**

Li-Ba Gei et al.

*Corresponding author: Dong-Tai Chen, chendt@sysucc.org.cn

**This PDF file includes:**

Supplementary Figures: Figs. S1 to S3

Supplementary Figures

**Fig. S1. Zonisamide and morphine do not affect BV-2 cell viability.**

BV-2 cells were treated with morphine (200 μM) and/or zonisamide (10 μM) for 12 h. Cell viability was assessed using the CCK-8 assay and expressed as a percentage of the control group. Data are presented as mean ± SD (n = 4). One-way ANOVA followed by Bonferroni's post-hoc test revealed no significant differences among groups (P > 0.05).

**Fig. S2. Baseline nociceptive thresholds and TLR4 inhibitor (TAK-242) experiment.**

(A) Baseline paw withdrawal latencies were measured on days 1, 3, 5, 7, and 9 prior to morphine administration in the hot-plate test. No significant differences were observed among groups (two-way repeated-measures ANOVA followed by Bonferroni's post-hoc test, *P* > 0.05). Behavioral data were expressed as percentage of maximal possible effect (%MPE). (B, C) Time course of the analgesic response in the hot-plate (B) and tail-flick (C) tests over the 9-day treatment period. Mice were treated with saline, morphine (10 mg/kg, s.c.), morphine + zonisamide (30 mg/kg, i.p.), morphine + TAK-242 (3 mg/kg, i.p.), or morphine + TAK-242 + zonisamide. Data are expressed as percentage of maximal possible effect (%MPE). Two-way repeated measures ANOVA revealed a significant treatment × time interaction (B: F(16,80) =6.65, *P* = 2.87 × 10^-9^; C:F(16,80) =5.392, *P* = 1.47 × 10^-7^). Bonferroni post-hoc comparisons showed that all three treatment groups had significantly higher nociceptive thresholds than the morphine alone group at multiple time points. **P* < 0.05, ***P* < 0.01, ****P* < 0.001 versus the morphine group. (D, E) Area under the curve (AUC) analysis of the hot-plate (D) and tail-flick (E) tests over the 9-day period. Pharmacological inhibition of TLR4 with TAK-242 partially recapitulated the protective effect of zonisamide, and no additional benefit was observed when zonisamide was combined with TAK-242. Data are presented as mean ± SD (n = 6).One-way ANOVA followed by Bonferroni post-hoc test: (D: F(4,25) =313.7, *P* = 1.34 × 10^-22^; E：F(4,25) =80.84, *P* =6.3×10^-14^ ). **P* < 0.05, ***P* < 0.01, ****P* < 0.001 versus the morphine group. Abbreviations: Mor, morphine; ZNS, zonisamide.

**Fig. S3. Zonisamide attenuates morphine-induced astrocytic activation in the spinal cord.**

(A) Representative immunofluorescence images of GFAP staining in the spinal cord dorsal horn of the saline, morphine, zonisamide, and morphine + zonisamide groups. Scale bar = 50 μm. (B) Quantification of GFAP fluorescence intensity. Morphine significantly increased GFAP immunoreactivity, which was attenuated by zonisamide co-treatment. Data are presented as mean ± SD (n = 3). Statistical analysis was performed using one-way ANOVA followed by Bonferroni post-hoc test ( F(3, 8) = 75.49, *P* = 3.29× 10^-6^). ****P* < 0.001 versus the Saline group; ##*P* < 0.01 versus the morphine-treated group. Abbreviations: Mor, morphine; ZNS, zonisamide.
