# Supplementary material for: Zonisamide attenuates morphine tolerance in association with reduced spinal TLR4/p38 MAPK signaling
Source: Front Pharmacol. 2026 Aug 26;17:1893236. doi: 10.3389/fphar.2026.1893236 (PMC13553760; doi:10.3389/fphar.2026.1893236)
Supplement: Supplementary file 2 [file Supplementaryfile2.docx]

Supplementary Materials

Fig.1-G

P-p38-1(control/mor/ZNS+Mor/ZNS)	p38-1	GAPDH

P-p38-2(control/mor/ZNS+Mor/ZNS)	p38-2	GAPDH

P-p38-3(control/mor/ZNS+Mor/ZNS)	p38-3	GAPDH

P-p38-4 (control/mor/ZNS+Mor/ZNS)	p38-4	GAPDH

Fig.3-A

Iba-1-1(saline/mor/ZNS+Mor/ZNS)	GAPDH

Iba-1-2(saline/mor/ZNS+Mor/ZNS)	GAPDH

Iba-1-3(saline/mor/ZNS+Mor/ZNS)	GAPDH

Iba-1-4 (saline/mor/ZNS+Mor/ZNS)	GAPDH

Fig.4-A

TLR4-1(saline/mor/ZNS+Mor/ZNS)	GAPDH

TLR4-2(saline/mor/ZNS+Mor/ZNS)	GAPDH

TLR4-3(saline/mor/ZNS+Mor/ZNS)	GAPDH

TLR4-4(saline/mor/ZNS+Mor/ZNS)	GAPDH

Fig.4-B

P-p38-1(saline/mor/ZNS+Mor/ZNS)	p38-1	GAPDH

P-p38-2(saline/mor/ZNS+Mor/ZNS)	p38-2	GAPDH

P-p38-3(saline/mor/ZNS+Mor/ZNS)	p38-3	GAPDH

P-p38-4(saline/mor/ZNS+Mor/ZNS)	p38-4	GAPDH

Fig.4-C

IL-1β-1(saline/mor/ZNS+Mor/ZNS)	GAPDH

IL-1β-2(saline/mor/ZNS+Mor/ZNS)	GAPDH

IL-1β-3(saline/mor/ZNS+Mor/ZNS)	GAPDH

IL-1β-4(saline/mor/ZNS+Mor/ZNS)	GAPDH

Fig.4-D

TNF-α-1(saline/mor/ZNS+Mor/ZNS)	GAPDH

TNF-α-2 (saline/mor/ZNS+Mor/ZNS)	GAPDH

TNF-α-3(saline/mor/ZNS+Mor/ZNS)	GAPDH

TNF-α-4(saline/mor/ZNS+Mor/ZNS)	GAPDH
